# Molecular understanding of wood formation in trees

**DOI:** 10.48130/FR-2022-0005

**Published:** 2022-04-25

**Authors:** Laifu Luo, Laigeng Li

**Affiliations:** 1 National Key Laboratory of Plant Molecular Genetics and CAS Center for Excellence in Molecular Plant Sciences, Chinese Academy of Sciences, Shanghai 200032, China; 2 University of the Chinese Academy of Sciences, Beijing 100049, China

**Keywords:** Wood formation, Vascular cambium, Cell expansion, Secondary cell wall, Secondary xylem

## Abstract

Trees convert and store the majority of their photosynthetic products in wood which is an essential renewable resource much in demand by human society. Formation of wood follows a process of consecutive cell developmental stages, from vascular cambium proliferation, cell expansion and differentiation, secondary cell wall deposition to programmed cell death, which is controlled by the functionality of complex molecular networks. What are the molecular networks involved in wood formation? How do the molecular networks act in a way to generate wood tissue during tree growth? What are the regulatory modules that lead to the formation of various wood characteristics? The answers to these questions are fundamental to understanding how trees grow, as well as how we can genetically engineer trees with desired properties of wood for human needs. In recent years, a great deal of interest has been invested in the elucidation of wood formation at the molecular level. This review summarizes the current state of understanding of the molecular process that guides wood formation in trees.

## Introduction

Trees are major constituents of terrestrial ecosystems and every year more than half of the carbon fixed by terrestrial plants can be attributed to trees^[1]^. Most of the carbon fixed in trees is stored in wood, therefore, wood is an important carbon reservoir and plays an important role in the balance of oxygen and carbon dioxide in the atmosphere. In addition, as a natural renewable resource, wood is widely used in human society, such as in pulping, construction, lignocellulosic biofuels, and other wood-based chemicals. Understanding how wood is produced through complex molecular processes has tremendous significance in maximizing carbon storage in trees and is also essential for the genetic improvement of trees for wood production.

**Figure 1:**
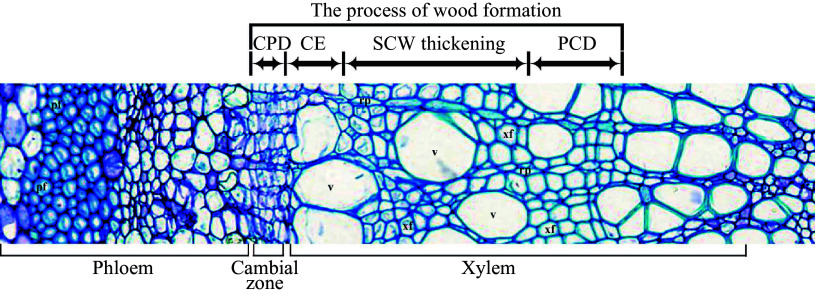
The cross-section of tree trunk shows the wood formation process. CPD, cell proliferation/differentiation; CE, cell expansion; SCW thickening, secondary cell wall thickening; PCD, programmed cell death; v, vessel; xf, xylem fiber; rp, ray parenchyma cell, pf, phloem fiber.

Formation of wood (secondary xylem) is a sequential developmental process that starts from the vascular cambium, via cell division/differentiation, cell expansion, secondary cell wall (SCW) deposition and programmed cell death (PCD) (Fig. 1) to form wood tissue. The different developmental stages involve a distinct battery of genes to carry out various molecular and cellular activities in directing wood cell development^[2]^. Here we try to summarize the current understanding of the molecular processes that occur in wood formation of trees.

## Molecular basis for vascular cambium proliferation and xylem differentiation

Vascular cambium, derived from procambium in the vascular bundle, is a ring of meristem tissue which grows the stem girth. Vascular cambium cell proliferation produces daughter cells that differentiate into various cell types through different pathways. Generally, the daughter cells differentiate outward into secondary phloem and inward into secondary xylem (wood tissue). Wood tissue is mainly composed of tracheids and parenchyma cells in gymnosperms, but vessels, fibers and parenchyma cells in angiosperms, which provide mechanical support and long distance transportation of water and minerals for upright growth^[3]^. The vascular cambium proliferation and differentiation are programmatically carried out and precisely regulated through complex signals and molecular networks.

Phytohormones play an essential role in controlling vascular cambium activities. Of them, auxin, cytokinin and brassinosteroids are known to be the main phytohormones involved in regulating cambium proliferation activity. Auxin regulates cell fate to promote xylem identity^[4]^. In trees, auxin is distributed in a bell curve of concentration across the phloem-cambium-developing xylem area, peaking in the cambium district^[5,6]^. It is postulated that the gradient of auxin concentration affects cambium proliferation and differentiation^[7]^. Regulation of the auxin concentration modifies cambium activity and wood formation. In *Populus*, two vascular cambium-related MADS-box genes, *VCM1* and *VCM2*, are found to modulate the soluble auxin concentration in cambium cells by regulating expression of *PIN5*, thereby regulating vascular cambium activity^[8]^. *VCM1* and *VCM2* bind to the *PIN5* promoter to enhance *PIN5* expression, while PIN5, localized in the ER membrane, transports active IAA from the cytosol to the ER lumen, in which the active IAA is deactivated. Thus, the *VCM1/VCM2*-*PIN5* functions as a molecular module to tune the auxin homeostasis in the vascular cambium district and hence to modify the vascular cambium activity. Auxin levels can trigger molecular cascades through auxin early response genes, such as the *Auxin/Indole-3-Acetic Acid* (*Aux/IAA*) family and *Auxin Response Factor* (*ARF*) family. *PttIAA3* is a *Populus IAA* member predominantly expressed in the cambial meristem and developing secondary xylem cells^[9]^. By upsetting the auxin signaling through expression of a dominant mutant version of *PttIAA3*, the cambium cell division activity is reduced, affecting the secondary xylem development with little effect on phloem development^[10]^. *PtoIAA9*, another *IAA* gene in *Populus*, is also preferentially expressed in cambium and its neighboring cells^[11]^. PtoIAA9 can interact with PtoARF5 to regulate cambium division and xylem development via orchestrating the expression of the HD-ZIP III transcription factor genes *PtoHB7*/*8* which correlate with cambium proliferation and xylem cell differentiation in poplar^[11]^. There are a total of 35 *Aux/IAA* and 39 *ARF* genes in the *Populus* *trichocarpa* genome^[12]^. It is unclear how many *Aux/IAA*s and *ARF*s are specifically involved in auxin signaling in the regulation of wood formation. Nevertheless, the experimental evidence shows that auxin and its signaling play a key role in the regulation of cambium activity and secondary xylem development. However, the knowledge regarding how auxin signaling is conducted in cambium cells to direct cell proliferation and differentiation is far from complete. Many questions remain to be studied, such as how auxin determines wood cell type differentiation? What are the molecular pathways, by which the auxin signaling is transduced downstream of the ARFs to cell proliferation, differentiation and SCW formation?

Cytokinin (CK) is another phytohormone known to play a role in wood formation. CK is distributed across the vascular cambium area with its peak concentration in the cells of the developing phloem side next to the cambium^[6]^. CKs can stimulate cambium cell division and thus affect wood formation^[13]^. Reducing the CK level by overexpression of a cytokinin catabolic gene, the *Arabidopsis CYTOKININ OXIDASE 2* (*CKX2*) gene in poplar, results in inhibition of the radial growth, likely due to the decrease of cell division in vascular cambium^[13]^. The spatial distribution of CK and auxin in the cambium zone is partially overlapped^[6]^. Increase of CK biosynthesis in the cambial zone and developing xylem cells result in an increase in auxin concentration in the cambial zone^[6]^, suggesting that the two hormone signals are interconnected in regulating cambium proliferation and differentiation. However, the molecular pathways underlying the crosstalk of the two hormones as it relates to control of wood formation are yet to be studied. A recent study showed that reduction of the CK level in phloem cells by phloem-specific expression of *cytokinin oxidase/dehydrogenase* *2* (*CKX2*, a gene encoding a cytokinin degrading enzyme), restricts the cambial proliferation activity^[14]^. This suggests that CK is transported from phloem to cambium cell for directing cell division.

Brassinosteroids (BRs) are shown to be related to cambial activity and it may coordinate with IAA to promote cambium cell division^[15]^. In *Populus*, exogenous application of 24-epi-brassinolide (BL, an active form of BRs) results in enhancement of cambial cell division while application of propiconazole (PCZ, an inhibitor of BR biosynthesis) leads to inhibition of cell division^[16]^. Additionally, other phytohormones such as ethylene^[17,18]^, gibberellins^[19]^ and abscisic acid^[20]^, are also reported to be involved in modulating cambium proliferation and cell differentiation in trunk growth of trees. However, it is unclear how signal transduction of these hormones is carried out in cambium and developing xylem cells. A more challenging question yet to be answered is how the multiple phytohormone signals are mutually connected in regulating vascular cambium proliferation and differentiation during tree growth.

In addition to traditional phytohormones, small peptides play a critical role in modulating cambial activity. The CLAVATA3 (CLV3)/EMBRYO SURROUNDING REGIONRELATED (CLE) peptide is recognized by its receptor to regulate the molecular networks involved in the maintenance of meristem identity and activity in *Arabidopsis*^[21,22]^. Activity of the vascular cambium in trees is also regulated by CLE peptides. The *Populus* homologs of *CLE41* and *PHLOEM INTERCALATED WITH XYLEM* (*PXY*) play a role in directing cambium division^[23]^. Overexpression of *PttCLE41* that encodes a peptide ligand known as TRACHEARY ELEMENT DIFFERENTIATION INHIBITORY FACTOR (TDIF)^[24]^ and its receptor kinase gene *PttPXY* in hybrid aspen results in increased cambial cell division^[23]^. Meanwhile, the *PttWOX4* (*WUSCHEL HOMEOBOX RELATED 4*) gene, a transcription factor downstream of the CLE-PXY signaling, is speciﬁcally expressed in the cambium region. Suppression of *PttWOX4* leads to reduction of secondary growth in trees^[25]^. The studies suggest that CLE-PXY-WOX4 forms a molecular module in promoting vascular cambium activity. Meanwhile, other CLE genes, such as *PtrCLE20* and *PttCLE47* are also found to play a role in the regulation of the vascular cambium activity in *Populus*^[26,27]^. *PtrCLE20* is transcribed in developing xylem and its derived peptide displays inhibition on cambium cell dividing activity. Likely the peptide is moved from developing xylem to cambium cells where it inhibits vascular cambium proliferation^[26]^. It is proposed that the cambium activity may be coordinately regulated by a pair of peptide signals respectively from the phloem and xylem^[26]^. The CLE41 peptide, which is originated from phloem cells^[24,28,29]^ to deliver a positive signal and the CLE20, which is originated from xylem cells to convey a negative signal^[26]^, meet in the cells of the vascular cambium to regulate cambium proliferation in a synchronized manner. This proposition projects a molecular view of how wood formation is tuned by the signals from two opposite locations. Nevertheless, more analysis would provide multi-faceted evidence for further corroboration.

In an analysis of transcriptional activity in wood formation, high-spatial-resolution RNA sequencing identified a group of transcription factor genes actively expressed in the cambium-xylem area^[30,31]^. Although many of the transcription factor genes expressed in the wood-forming tissue are yet to be studied for their function in cambium activity and xylem differentiation, several have been characterized for their roles in regulating wood formation in trees. The *HD-ZIP III* gene family has been shown to regulate vascular patterning, differentiation of cambium daughter cell, polar auxin transport, organ polarity in *Arabidopsis*, such as *ATHB*-*8, PHV*/*ATHB*-*9*, *CORONA*/*ATHB*-*15*, *PHABULOSA* (*PHB*)/*ATHB-14*, and *INTERFASCICULAR FIBERLESS1* (*IFL1*)/*REVOLUTA* (*REV*)^[32−34]^. The *P. trichocarpa* genome contains seven members of the *HD-ZIP III* gene family but five in *Arabidopsis*. *HD-ZIP III* genes are highly expressed in relation to vascular cambium development and xylem differentiation^[35−38]^. Among them, alteration of *PopREVOLUTA* (*PRE*, an orthologue of *Arabidopsis* *IFL1/REV*.) expression in *Populus* leads to defects in vascular tissue patterning^[36]^. Mis-expression of *PopCORONA* (*PCN*), a homologous gene of the *Arabidopsis* *Corona*/*ATHB-15*, results in defects of xylem cell differentiation^[37]^. *PtrHB7*, the homologous gene of *ATHB-8*, is highly expressed in cambium and functions in balancing the differentiation between secondary xylem and phloem tissues^[35]^. Suppression of *PtrHB7* results in enhanced differentiation of cambium cells to phloem but has an inhibitory effect to xylem, while *PtrHB7-*overexpressed plants display the opposite phenotype^[35]^. Furthermore, *PtrHB4* is required for development of the interfascicular cambium to form the vascular cambium in trees^[38]^. These studies indicate that the *HD-ZIP III* members act in different stage of wood formation, from vascular patterning, vascular cambium formation and xylem differentiation. In response to auxin signaling, *HB7/8* are regulated by the IAA9-ARF5 module in cambium proliferation in *Populus*^[11]^. It appears that the HD-ZIP III regulators play a key role in cambium proliferation and differentiation. Full characterization of the HD-ZIP III mediated pathways would provide more insights into understanding wood formation.

The transcriptional regulatory networks during wood formation may be initially orchestrated by phytohormones as fundamental signaling. In *Populus*, *PaC3H17* and *PaMYB199* expression is regulated by auxin, and *PaC3H17* and *PaMYB199* display opposite roles in cambium cell proliferation^[39]^. *PaMYB199* plays a repressive role in cambium proliferation while its expression is inhibited by *PaC3H17*. Conceivably, PaC3H17 and PaMYB199 may act as intermediate players in the auxin signaling pathway to regulate cambium proliferation^[39]^.

**Figure 2:**
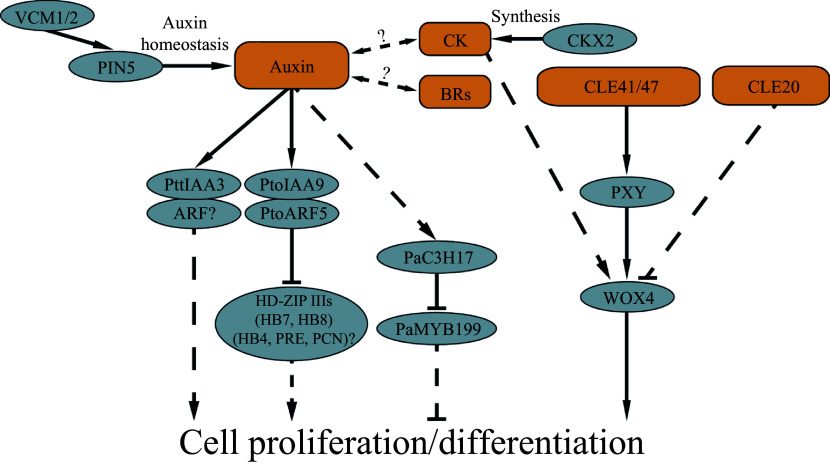
Signal molecules and molecular networks identified in cambial cell proliferation and differentiation of trees.

Vascular cambium development, proliferation and subsequent cell differentiation are primary processes in cellular development of wood formation in trees. To date, studies have shown that phytohormones, peptides and molecular modules play various roles in regulating cambium proliferation and differentiation (Fig. 2). This knowledge represents a foundation toward a more comprehensive understanding of the molecular mechanisms underlying wood formation in trees.

## Molecular process of cell expansion in wood formation

After the secondary xylem mother cells are produced from cambium cells, they undergo cell expansion, followed by deposition of the SCW, lignification and cell death to form wood cells (Fig. 1). The cell expansion process determines the final cell shape and size in wood. This process mainly occurs during primary cell wall formation and modification. The primary cell wall is composed of 20%−30% cellulose, 30%−50% pectin, 20%−25% hemicelluloses and about 10% glycoproteins^[40]^. Cellulose and hemicellulose form a network structure embedded in pectin^[41]^. Both disrupting the connection between cellulose and hemicellulose or breaking the hemicellulose can disperse the cellulose microfibrils to promote the loosening of the cell wall. Multiple enzymes are identified as primary players in this process.

A series of genes encoding enzymes related to cell wall modification in *Populus* stem have been identified, such as *EXPANSIN*s, *XTHs*, *PMEs*, *PL1s*^[42−44]^. EXPA (α-expansin) and EXPB (*β*-expansin) can bind to xyloglucan and xylose, respectively. It is speculated that they may untie the non-covalent bonds between xyloglucan/xylose and other cell wall components to promote cell wall loosening^[45]^. Exogenous applications of expansins to cell walls can quickly induce cell wall extension without affecting its plasticity and flexibility^[46]^. A number of *expansin* genes are expressed in the radial expansion zone of *Populus* stem, such as *EXPA1*, *EXPA5*, and *EXPA7*^[42,47]^. Overexpression of *PttEXPA1* in hybrid aspen resulted in increase of stem internode length and fiber diameter growth^[48]^. Furthermore, xyloglucan endotransglucosylase (XET)/hydrolase (XTH) are involved in cell wall loosening and remodeling, possibly by catalyzing the hydrolysis and reconnection of xyloglucan^[49]^. In *Populus*, multiple *XTHs* are highly expressed in wood-forming tissues^[42,50]^. Developing gelatinous (G) layer in xylem fiber cells in *Populus* is related to the XET activity^[51]^. Overexpression of *PtxtXET16-34* showed an increase of the vessel diameter and change of xyloglucan content^[50]^.

In addition to expansins and XTH, glycosyl hydrolases also participate in cell wall loosening, such as endo-1,4-*β*-glucanases and endo-1,4-β-mannanase. Endo-1,4-*β*-glucanases (EGases) is thought to function in cell wall loosening through its hydrolyzing xyloglucans activity^[52]^. *PopCel1* and *PopCel2* encode two EGases. Overexpression of these two genes promotes plant growth by enhancing cell expansion^[53]^. When *PtrCel9A6* and *PtrGH9A7* from *Populus* are expressed in *Arabidopsis*, the transgenics showed an increase in plant growth and fiber cell length, suggesting that EGases are applicable to the modification of plant growth and cell length^[54,55]^. Another hydrolase, endo-1,4-β-mannanase hydrolyzes mannan-type polysaccharides to generate oligosaccharides. Overexpression of *PtrMAN6*, an endo-1,4-β-mannanase gene in *Populus*, leads to enhanced cell wall loosening but inhibits cell wall thickening, while knockdown of its expression has the opposite effect^[56]^. The PtrMAN6 function in coordinating cell wall remodeling and thickening may be mediated through oligosaccharide signals^[56]^.

Pectin is a major component of the primary cell wall, critical to remodeling and expansion of cell walls. Pectin methyl esterases (PMEs) and pectin acetylesterases (PAEs) are two enzymes of pectin modification to control cell wall loosening^[57,58]^. Modification of *PME1* in *Populus* affects expansion of the wood cells and results in changes in fiber cell length and width^[59]^. High PME activity is detected in the cambium and developing xylem region in *Populus* stem, suggesting that PME is important for the regulation of dynamic cell expansion during wood formation^[60]^. Overexpression of the *Populus trichocarpa PAE1* gene in tobacco reduces the degree of pectin acetylation and alters cell wall characteristics^[58]^.

Hormone signals may play roles in cell elongation and expansion. Overexpression of GA biosynthesis-related genes such as *GIBBERELLIN 20-OXIDASE 1* (*GA20ox1*) in transgenic *Populus* increases the endogenous GA content and promotes elongation of xylem fibers and growth^[61,62]^. Moreover, studies have shown that overexpression of the ethylene response factor gene *ERF139* in *Populus* reduced the diameter of vessels while the *ERF139* dominant negative mutant displayed an increase in vessel diameter^[63]^. Ethylene can induce the expression of *ERF139* and *ERF118*^[64]^. *ERF118* in *Populus* is reported to be associated with cell expansion^[65]^. These results suggest that ethylene signaling may be involved in the cell expansion of wood. Auxin signaling is involved in promoting cell expansion^[66]^, however, the specific mechanism regulating the role of auxin in wood cell expansion is not fully understood.

As discussed above, xylem cell expansion in the process of wood formation is regulated by many factors, including plant hormones, expansins, hydrolases, and other molecular networks. The molecular knowledge underlying xylem cell expansion would have implications in tree improvement.

## Transcriptional networks in wood secondary cell wall deposition

After cell expansion, SCW deposition is initiated. SCW is the main biomass produced by land plants and stores most of the carbon fixed by photosynthesis. In trees, SCW is primarily deposited in xylem tissue, i.e. wood. SCW are mainly composed of three kinds of polymers, including 60% cellulose, 10%−40% hemicellulose and 30% lignin^[67]^. Biosynthesis and assembly of SCW during wood formation is regulated by a battery of elaborate transcriptional networks.

**Figure 3:**
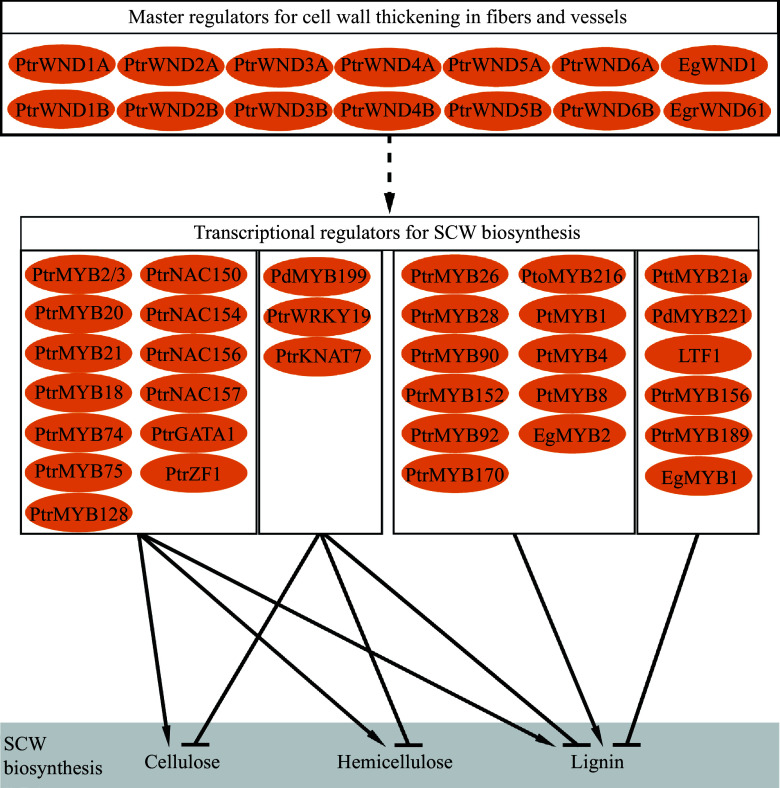
Transcription factor genes in regulation of wood secondary cell wall biosynthesis.

Biosynthesis of SCW involves multiple metabolic pathways to convert photosynthetic products into three major biopolymers. Coordination of the biosynthesis and assembly of the biopolymers to deposit in SCW during wood formation is strictly regulated through hierarchical transcriptional networks (Fig. 3). The regulatory networks are constructed in multiple layers composed of NAC and MYB transcription factors^[68−71]^. In *Arabidopsis*, a number of NAC transcription factors such as Vascular-related NAC-domain1 (VND1) to VND7, NAC secondary wall thickening promoting factor 1 (NST1), and secondary wall-associated NAC domain protein 1 (SND1/NST3) and NST2 constitute the top-level master regulators to regulate the expression of other downstream TFs and genes related to wall synthesis^[72−74]^. In tree species, the compeer master regulators in the hierarchical networks are named WNDs (wood-associated NAC domain transcription factors)^[75,76]^. Compared to *Arabidopsis*, the *Populus* genome contain more homologous genes of the master regulators. For example, there are three *NST* homologs (*NST1*, *NST2* and *NST3/SND1*) in *Arabidopsis,* and four homologous genes (*WND1A, WND1B, WND2A* and *WND2B*) in *Populus* *trichocarpa* which function differently^[75,77,78]^. *PtrWND1B*, an ortholog of *Arabidopsis* *SND1*, functions in fiber cell wall thickening. Suppression of *PtrWND1B* expression speciﬁcally inhibited ﬁber cell wall thickening^[78]^. *PtrWND1B* alternative splicing produces two isoforms which play antagonistic roles in regulating fiber cell wall thickening, which does not occur in *Arabidopsis* and may act as a particular mechanism to regulate xylem fiber cell wall thickening in trees^[78,79]^. Overexpression of the normal short-transcript *PtrWND1B-s* enhanced fiber cell wall thickening, while overexpression of the alternative long-transcript *PtrWND1B-l* repressed ﬁber cell wall thickening^[78]^. Meanwhile, *EgWND1*, an *SND1* homolog in *Eucalyptus*, also shows its relation to SCW deposition. Its overexpression in *Arabidopsis* causes ectopic deposition of SCW^[80]^. Modification of *PtrWND2B* and *PtrWND6B* expression in *Populus* results in changes to SCW thickening and biosynthesis of lignin and cellulose^[77,81]^. *WND*s act as main transcriptional regulators to control wood SCW formation through a set of hierarchical networks in trees.

WNDs activate downstream genes through its binding to the secondary wall NAC-binding element (SNBE) in the promoter of target genes^[81,82]^. In *Populus*, the *PtrMYB3* and *PtrMYB20* promoters contain multiple SNBE elements that are targeted by PtrWNDs^[81]^. A number of the MYB transcription factors constitute a complex of regulatory networks downstream of the NAC transcription factors to hierarchically regulate SCW formation in response to internal developmental signals and environmental stimuli^[68,69,81]^. In *Arabidopsis*, MYB46 and MYB83 are the second layer master switches in the regulatory network, which can be directly targeted by NAC to activate the secondary wall biosynthetic program^[83,84]^. *PtrMYB2*/*21* and *PtrMYB3*/*20*, the homologous genes of *AtMYB46* and *AtMYB83* in *Populus*, respectively, which are activated by WNDs to regulate the biosynthesis pathways of cellulose, lignin and hemicellulose^[85,86]^. Studies also show that *PtrMYB18*/*74*/*75*/*128* are also able to activate the expression of SCW biosynthesis genes^[81]^. In addition, several regulators have been identified for specific regulation of lignin biosynthesis. *PtMYB4* and *EgMYB2* are homologous genes of *AtMYB46/83* in pine and *Eucalyptus*, respectively, and constitutive overexpression of these genes induces ectopic SCW formation, particularly lignin biosynthesis^[87,88]^. Similarly, *PtrMYB26*/*28*/*90*/*152* in *P. trichocarpa*^[81,89,90]^, *PtoMYB92*/*170*/*216* in *P. tomentosa*^[91−94]^, and *PtMYB1*/*8* in pine^[95]^ are reported to specifically regulate lignin biosynthesis. On the other hand, SCW biosynthesis can be regulated through repression mechanisms. *PdMYB199*, a homologous gene of *AtMYB42/85*, plays a negative role in SCW thickening by inhibiting expression of SCW biosynthesis genes^[39]^. In *Populus*, *PttMYB21a*^[96]^, *PdMYB221*^[97]^, *PtoMYB156*^[98]^, *PtrMYB189*^[99]^ and *Eucalyptus* *EgMYB1*^[100,101]^ are transcriptional repressors inhibiting lignin biosynthesis. *LTF1*, one of the *AtMYB4* homologous genes in *Populus* acts as a sensory switch to specifically repress lignin biosynthesis^[102]^.

Furthermore, expression of *EgrNAC61*, a *SND1* homolog in *Eucalyptus*, is positively related to wood SCW formation and displays activity in *Arabidopsis* protoplast to induce expression of the SCW biosynthesis genes^[103]^. *PtrNAC154*^[104]^, *PtrNAC150*/*156*/*157*, *PtrZF1* and *PtrGATA1* (two zinc-ﬁnger transcription factors)^[81]^ are identified in *Populus* as activators for SCW thickening, while *PtrWRKY19* and *PtrKNAT7*^[105,106]^ exert repression on expression of cellulose, hemicellulose and lignin biosynthesis genes.

Studies have revealed a complex of regulatory genes that control formation of SCW in tree wood (Fig. 3). Generally, the identified genes display regulatory roles similar to their homologs in herbaceous model species. However, many of them perform distinct functions specific to tree species. For example, alternative splicing of *PtrWND1B* illustrates a mechanism to sustain homeostatic fiber cell wall thickening during wood formation in *Populus*, while its orthologs in *Arabidopsis* do not undergo alternative splicing^[78]^. In terms of cell wall structure and chemical composition, wood SCWs show diverse characteristics in various tree species and are also very different from those in herbaceous species^[107,108]^. Thus, formation of the characteristic of wood SCWs may be attributed to specific regulations unique to tree species, which are largely unknown. Understanding of the regulations underlying wood SCW formation has huge implications for tree improvement for biomaterial production.

## Biosynthesis of secondary cell wall

SCW is mainly composed of cellulose, hemicelluloses and lignin, which are also the main components of wood^[3]^ . To date, most of the genes that encode the enzymes catalyzing SCW biosynthesis during wood formation have been characterized.

Cellulose, a biopolymer of unbranched *β*-(1,4)-linked glucan chains, is the most abundant component in wood SCW and its content has a substantial effect on its properties. Cellulose biosynthesis is catalyzed by cellulose synthases (CESAs)^[109]^, which form a cellulose synthesis complex (CSC) localized on the plasma membrane (PM). On the PM, CSCs are co-localized with cortical microtubules (MTs) and moves along the MT bands to catalyze glucan chain elongation to form cellulose microfibrils extending into cell walls^[67,110]^. The *P. trichocaropa* genome contains 18 *CESA* genes to form two types of CSCs to synthesize cellulose wood cell walls^[111,112]^. Knockout or suppression of *CESA* in *Populus* results in the disruption of the SCW layered structure as well as alteration of the cellulose content and structure^[113,114]^. The CESA in different types of CSC showed distinct effects on the cellulose microfibril structure^[114]^. This suggests a possibility that CESA composition in CSC affects formation of the cellulose microfibril structure. In addition, wood cellulose biosynthesis involves other proteins. *Ptrcel9A6* and *PtrKOR1* (*KORRIGAN 1*) are EGase genes which are required for SCW cellulose formation^[55,115]^. They are expressed in developing xylem undergoing secondary wall thickening. Suppression of their expression results in thinner SCW with reduced cellulose content^[55,115]^. Studies have shown that sucrose synthase (SUS) is involved in cell wall thickening and cellulose biosynthesis^[116,117]^. While in tree species, reduction of the SUS activity does not specifically affect cellulose content but causes an overall decrease of biopolymers in wood^[118]^, indicating that SUS may not be particularly involved in cellulose biosynthesis. The CYTOSOLIC INVERTASEs (CINs) convert sucrose to UDP- glucose. Suppression of CIN activity in *Populus* leads to a decrease in UDP- glucose and cellulose^[119]^.

Cellulose is a main component of wood and there has been a great deal of investigation to understand cellulose biosynthesis and its regulation. While the general molecular mechanism of cellulose biosynthesis has been elucidated, much more of its regulation is yet to be studied. Full understanding of how cellulose biosynthesis can be manipulated in relation to wood properties would help to engineer trees with desired cellulose and wood characters.

Hemicellulose is a heteropolysaccharide and can form linkages with cellulose and lignin to make cell wall polymers connect together^[120]^. There are different types of hemicelluloses, such as xyloglucan, xylan, mannan, and *β*-(1→3)(1→4)-glucan. Xylan and mannan are the main hemicelluloses in SCW^[121]^.

Xylan is composed of a β-(1,4)-xylan backbone decorated with glycosyl substitutions and modifications with methyl and acetyl groups^[122]^. Genetic and biochemical analyses revealed that *β*-1,4-xylan backbone biosynthesis is mediated mainly by a xylan synthase complex composed of IRX10/IRX10L, IRX9/IRX9L and IRX14/IRX14L. Mutations in these genes show defects in xylan synthesis^[122−127]^. In *Populus*, five GT43 glycosyltransferases are encoded by *PtrGT43A/B/C/D/E* genes, which are highly expressed in developing xylem^[128]^. Modification of their expression disrupts xylan synthesis^[128]^. In addition, more glycosyltransferase genes are reported to play a role in xylan synthesis. PoGT47C, PoGT8D and PoGT8E/F are suggested to act in synthesis of the xylan reducing end^[128−130]^. In wood xylan, glucuronic acid (GlcA) is often methylated at the O-4 position by glucuronoxylan methyltransferases (GXMs)^[131,132]^. PtrGXM1, PtrGXM2, PtrGXM3, and PtrGXM4 in *Populus* have been reported to mediate the methylation of GlcA in xylan during wood formation^[133]^. DUF579 proteins show diverse function and PtrUF579-3 is able to catalyze the GlcA methylation of xylan in *Populus*^[134,135]^. Suppression of *PtrDUF579-3* leads to a reduction in both the number of GlcA side chains and the degree of methylation on the GlcA side chain^[134]^. In addition to methylation, acetylation is another form of xylan modification. PtrXOATs (XYLAN O-ACETYLTRANSFERASE), DUF231 proteins in *Populus*, shows a catalyzing activity of acetylation on xylan^[136]^. The *REDUCED WALL ACETYLATION* (*RWA*) genes may play a role in the regulation of xylan acetylation. Four *RWA* genes in *Populus*, including *RWA-A*/*-B*/*-C*/*-D*, are expressed in developing wood and downregulation of these genes result in a reduction of xylan and xyloglucan acetylation in wood^[137]^.

Glucomannan and galactoglucomannan are two types of mannan SCWs. Glucomannan is mainly formed in angiosperms while galactoglucomannan is a major hemicellulose in gymnosperms^[107]^. Biosynthesis of the β-1,4-glucomannan backbone is catalyzed by cellulose synthase-like family A (CslA)^[138]^. *Populus* *PtrCslA1* is highly expressed in wood-tissue, exhibiting glucomannan synthase activity for the biosynthesis of glucomannan during wood formation^[111]^.

Lignin, the second most abundant component after cellulose in wood, is a cross-linked phenolic polymer. Generally, lignin, which contains three subunits, H-, G- and S-monolignols, are polymerized by oxidative coupling of p-coumaryl alcohol, coniferyl alcohol and sinapyl alcohol^[2,108]^. Monolignol biosynthesis is achieved through a general phenylpropanoid pathway in cytosol and monolignols needed to be transported across the plasma membrane into the cell wall for polymerization. In the phenylpropanoid pathway, monolignol synthesis starts with deamination of phenylalanine catalyzed by phenylalanine ammonia-lyase (PAL), and is then catalyzed by a series of enzymes including cinnamate 4-hydroxylase (C4H), 4-coumarate:CoA ligase (4CL), Hydroxycinnamoyl-Coa Shikimate/Quinate Hydroxycinnamoyl Transferase (HCT), Caffeoyl-CoA O-methyltransferase (CCoAOMT), Cinnamoyl-CoA reductase (CCR), Ferulate 5-hydroxylase (F5H), Caffeate O-Methyltransferase (COMT) and Cinnamyl alcohol dehydrogenase (CAD), to form three monolignols^[108]^. Analysis of genes associated with the phenylpropanoid pathway in *Populus* show a set of 23 genes that encode for the monolignol biosynthesis enzymes. Eighteen of the genes are preferentially expressed in developing xylem and may play a core role in the biosynthesis of monolignols during wood formation^[139]^. During wood formation, changing the expression of these genes results in significant changes in the content of monolignols^[140]^. Meanwhile, alternative routes are reported in monolignols biosynthesis. For instance, caffeoyl shikimate esterase (CSE) can catalyze the conversion of caffeoyl shikimate to caffeic acid^[122]^. Modification of *CSE* expression in *Populus* leads to defective lignin biosynthesis^[141]^.

Monolignol polymerization is believed to be catalyzed by laccases (LACs) and peroxidases^[142]^. *Populus* genome contains 49 *LAC* genes and 17 of them are highly expressed in developing xylem, likely participating in lignin polymerization in a redundant manner^[143]^. *LAC* expression is regulated by Ptr-miR397a. Overexpression of *Ptr-MIR397a* in *Populus* results in reduction of the *LAC* transcript abundance and lignin content^[143]^. Studies show that peroxidase plays a role in cell wall lignification in *Arabidopsis*^[144]^ and down-regulation of anionic peroxidase alters both lignin content and composition in hybrid aspen^[145]^.

Lignin, a major component of SCW, is recalcitrant for the utilization of cell wall biomass in the production of biofuels and biochemicals^[146,147]^. Thus, modification of lignin is considered as an expectant strategy to circumvent the barrier for efficient processing of cell wall biomass^[148]^. Lignin modification in trees can change the lignin content and monolignol composition^[149−151]^. However, modification of lignin is often concomitant with defects in growth and development. For example, down-regulating the expression of 4CL in *Populus* results in a decrease in lignin content and is accompanied by stunting in the growth of transgenic *Populus*^[152]^. To overcome the growth penalty caused by lignin modification, recent studies have developed a new approach to engineer lignin through cell-type specific modifications. Cell-specific downregulation of *LTF1* or *4CL1* in the fibers and vessels of *Populus* xylem, respectively, result in the reduction of lignin content in fibers or vessels, while the fiber-specific lignin reduction does not affect plant growth but achieves improvements in wood biomass quality and saccharification efficiency^[153,154]^. In another study, vessel-specific reintroduction of lignin biosynthesis in the lignin biosynthesis-deficient *Populus* results in restoration of lignification in vessels and ray cells with hypoligniﬁcation in ﬁbers, and this modification yields increase of the wood biomass saccharification^[155]^. These studies indicate that lignin biosynthesis can be specifically regulated in different cell types in wood tissue. Cell-specific modification of lignin promises a new strategy to engineer lignin for improvement of wood cell wall biomass without growth penalty in trees.

## Molecular events in wood programmed cell death

In the process of wood formation, a series of irreversible autolysis, induced by the collapse of the vacuole, promotes PCD which is the last step in the formation of mature xylem cells^[156,157]^. Analyzing the maturation of secondary xylem of *Populus* revealed that many genes encoding proteases, nucleases, and autophage-related proteins are up-regulated before the PCD process of *Populus* xylem cells, indicating that they may be related to PCD during wood formation^[157]^. During the development of secondary xylem of *Populus*, 20S proteasome (20SP), a protein complex, was identified as having caspase-3-like activity related to PCD^[158]^. Treatments with Ac-DEVD-CHO (caspase-3 inhibitor) and clasto-lactacystin b-lactone (a proteasome inhibitor) inhibited the xylem differentiation of veins in *Arabidopsis* cotyledons and PCD of vessels in a VND6-induced Arabidopsis xylogenic culture, respectively, indicating the 20S proteasome is involved in xylem development and PCD^[158]^. *PttMC13* (*METACASPASES 13*) and *PttMC14* encoding cysteine proteases in *Populus* had been reported to play an important role in the process of proteolytic and xylem elements cell death^[159]^. Moreover, during PCD, endonucleases can catalyze the degradation of DNA, and two *Ca*^*2+*^*-dependent DNase* genes (*EuCaN1* and *EuCaN2*) encoding endonuclease were identified in secondary xylem of *Eucommia ulmoides*^[160]^. Disrupting their expression resulted in abnormal xylem development, which may be related to their role in PCD^[160]^.

Formation of characteristic heartwood in trees is an interesting physiological process. The longitudinal and radial parenchyma cells in some tree species remain alive over the course of several years to contribute to heartwood formation^[3,161,162]^. Clearly, those cells may not be normally programed to go to full PCD process but keep active in synthesis of the compounds needed for heartwood maturation^[3,161,162]^. The molecular mechanisms that control the process of heartwood formation need further investigation and research.

PCD is a crucial step in wood formation, accompanied by a series of irreversible hydrolysis processes and ultimate degradation of all cellular content, except the cell wall. While current understanding of PCD in relation to wood formation is insufficient, further research will help provide new insights into understanding of the development of distinct wood characteristics.

## Conclusions

With the continuous advancement of research in tree molecular biology, we are on the way to extending our knowledge of the molecular regulation of wood formation. Recently, the release of the cell atlas of *Populus* xylem obtained by single-cell sequencing^[163]^ provides a possibility to dissect the cellular developing lineage of wood formation at a single-cell resolution, which would open a new avenue to further our understanding of wood formation. Meanwhile, more and more tree genomes have being sequenced. Availability of the high-quality genome sequences which include tree species with a variety of wood properties will aid in mapping the genomic bases for regulation of the characteristics of wood formation. By combination of the genomic, genetic, molecular and cellular approaches, understanding of the molecular mechanisms underpinning wood formation will be advanced more quickly with more comprehensive elaborations, which will provide strong knowledge for improvement of wood with desired properties.
